# Consequences of Common Topological Rearrangements for Partition Trees in Phylogenomic Inference

**DOI:** 10.1089/cmb.2015.0146

**Published:** 2015-12-01

**Authors:** Olga Chernomor, Bui Quang Minh, Arndt von Haeseler

**Affiliations:** ^1^ Max F. Perutz Laboratories, Center for Integrative Bioinformatics Vienna, University of Vienna, Vienna, Austria.; ^2^ Bioinformatics and Computational Biology, Faculty of Computer Science, University of Vienna, Vienna, Austria.

**Keywords:** nearest neighbor interchange, partial terraces, phylogenetic terraces, subtree pruning and regrafting, tree bisection and reconnection

## Abstract

**In phylogenomic analysis the collection of trees with identical score (maximum likelihood or parsimony score) may hamper tree search algorithms. Such collections are coined phylogenetic terraces. For sparse supermatrices with a lot of missing data, the number of terraces and the number of trees on the terraces can be very large. If terraces are not taken into account, a lot of computation time might be unnecessarily spent to evaluate many trees that in fact have identical score. To save computation time during the tree search, it is worthwhile to quickly identify such cases. The score of a species tree is the sum of scores for all the so-called induced partition trees. Therefore, if the topological rearrangement applied to a species tree does not change the induced partition trees, the score of these partition trees is unchanged. Here, we provide the conditions under which the three most widely used topological rearrangements (nearest neighbor interchange, subtree pruning and regrafting, and tree bisection and reconnection) change the topologies of induced partition trees. During the tree search, these conditions allow us to quickly identify whether we can save computation time on the evaluation of newly encountered trees. We also introduce the concept of partial terraces and demonstrate that they occur more frequently than the original “full” terrace. Hence, partial terrace is the more important factor of timesaving compared to full terrace. Therefore, taking into account the above conditions and the partial terrace concept will help to speed up the tree search in phylogenomic inference.**

## 1. Introduction

In phylogenomics, one aims to reconstruct a phylogenetic *species* tree from multiple genes. One popular approach is to infer the trees from the concatenated gene alignment, the so-called supermatrix (Sanderson et al., 1998; De Queiroz and Gatesy, 2007). Here, if a gene sequence is not available for some taxon, it is represented by the sequence of unknown characters and is referred to as missing data. Several studies (van der Linde et al., 2010; Pyron and Wiens, 2011; Pyron et al., 2011; Nyakatura and Bininda-Emonds, 2012; Springer et al., 2012; Hedtke et al., 2013) use quite sparse supermatrices in their analysis and the percentage of missing data sometimes constitutes up to 95% (Peters et al., 2011).

Recently, it has been shown that missing data can hamper the tree search via existence of phylogenetic terraces (Sanderson et al., 2011), a collection of trees with exactly the same likelihood or parsimony score. Terraces occur in the analysis with *partitioned data*, that is, when distinct blocks of a supermatrix are treated differently (e.g., when each gene corresponding to one block evolves under its own evolutionary model). Two trees are said to belong to one terrace if the collections of their *induced partition trees* are exactly the same. Here, the induced partition tree is obtained by pruning the taxa on species tree, which have no sequence for the corresponding partition block.

Since the number of trees on one terrace can be quite large (Sanderson et al., 2011), accounting for terraces in tree search algorithms can potentially save a lot of computation time. During the tree search, one explores the tree space by moving from one candidate tree to another by means of topological rearrangements. If the topological rearrangement does not change any of the induced partition trees, then the two trees belong to the same terrace and a recomputation of objective function (maximum likelihood or maximum parsimony) used in the tree search is not necessary in order to evaluate a new tree.

Here, we first specify the conditions under which the topological rearrangements applied to the species tree change the corresponding induced partition trees. Using these conditions, one can quickly identify whether it is necessary to recompute the objective function for a given partition or not as a consequence of one of the three widely used rearrangements: nearest neighbor interchange (NNI), subtree pruning and regrafting (SPR) and tree bisection and reconnection (TBR) (Felsenstein, 2004).

We further generalize the concept of terrace to *partial terrace*, which is even more useful in practical phylogenetic analysis. We analyze several published alignments by examining NNI neighborhoods of random trees and trees encountered during the tree search using IQ-TREE (Nguyen et al., 2015). We show that for large number of taxa partial terraces are mainly determined by the missing data and less dependent on the actual tree topology analyzed. By taking into account partial terraces, it will be possible to speed up the tree search algorithms even in the absence of terraces.

The outline of the article is the following. We first introduce the notations and then discuss the important features of NNI, SPR, and TBR. Next, we specify the conditions when these topological rearrangements do not change the topology of induced partition trees. We further elucidate why such conditions are helpful even in the absence of terraces and define the concept of partial terrace. We analyze several published alignments to point out that partial terraces do occur in practice. Finally, we discuss the additional practical advantages of using induced partition trees in the maximum likelihood framework.

## 2. Background

### 2.1. Basic definitions and notations

In this section we provide basic definitions and notations used throughout the article. For a complete overview, see chapters 2, 3, and 6 in Semple and Steel (2003).

**Definition 2.1.** Let *X* be a taxon set. A *phylogenetic tree T* of *X* is a leaf-labeled tree with a bijection map from *X* into the set of leaves of *T*.

In the following, we work only with bifurcating phylogenetic trees; that is, all internal nodes have exactly three adjacent edges.

**Definition 2.2.** A *split*, denoted by *A*|*B*, is a bipartition of *X* into two nonempty, nonoverlapping sets *A* and *B*, where *A* ∪ *B* = *X*.

Note that *A*|*B* and *B*|*A* are equivalent. Every edge of *T* is associated with a split. When cutting an edge *e* of *T*, we obtain two subtrees with leaf labels *X*_1_ and *X*_2_, and then a split corresponding to *e* is defined as *X*_1_| *X*_2_. We denote this with *e* = *X*_1_| *X*_2_

We denote by *Σ*(*T*) a collection of all splits corresponding to edges of *T*.

The *symmetric difference* of two sets *A* and *B*, denoted *A*Δ*B*, is given by (*A*\*B*) ∪ (*B*\*A*), or the union of taxa present in *A* but not *B*, and vice versa.

**Definition 2.3.** Let *T*_1_ and *T*_2_ be the two leaf-labeled trees with the same label set *X*, and *Σ*(*T*_1_) and *Σ*(*T_2_*) be the collections of splits of *T*_1_ and, *T_2_*, respectively. Then the Robinson–Foulds (RF) distance (Robinson and Foulds, 1981) between *T*_1_ and *T_2_* is equal to |*Σ*(*T*_1_)Δ*Σ*(*T*_2_)|.

If for two trees the RF distance between them is 0, then they have the same collection of splits, and from splits-equivalence theorem (Semple and Steel, 2003; p. 43), the trees are *equivalent*.

**Definition 2.4.** Let *Y* be a subset of *X*. An *induced subtree* of *T*, denoted by *T*|*Y*, is a leaf-labeled tree with the following collection of splits:

\begin{align*}\Sigma ( T \mid Y ) = \{  A \cap Y \mid B \cap Y:
A \mid B  \in  \Sigma ( T ) \ { \rm and} \ A \cap Y \neq \emptyset
, B \cap Y \neq \emptyset \} .\end{align*}

For a species tree *T* and a given partition with taxon set *Y*, a *partition tree* is an induced subtree *T*|*Y*.

### 2.2. Topological rearrangement operations

In this section we introduce the topological rearrangements on trees commonly used in phylogenetic inference.

**FIG. 1.**
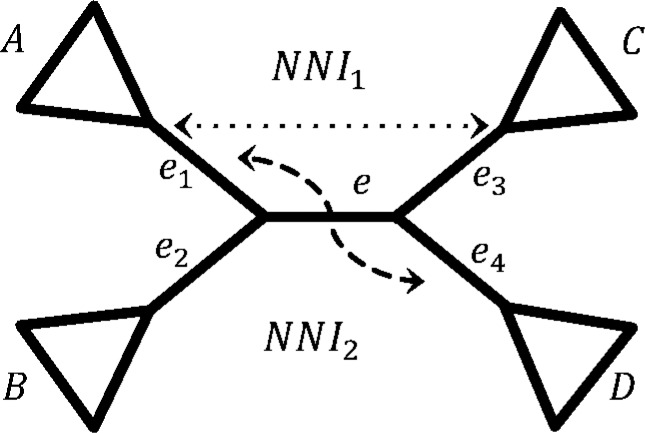
Visualization of NNI. Species tree *T* and the two NNIs around central edge *e. NNI*_1_ is obtained by exchanging subtrees below edges *e*_1_ and *e*_3_, while *NNI*_2_ by exchanging subtrees *e*_1_ and *e*_4_. NNI, nearest neighbor interchange.

The simplest possible operation that changes only one split on a tree is an NNI. It can only be applied to interior edges of the tree, since it requires the so-called quartet structure with an interior edge being the central edge of this structure (Fig. 1).

Let *e* be an interior edge of *T* and *e*_1_, *e*_2_, *e*_3_, *e*_4_ its four incident edges with *A*, *B*, *C*, *D* being the taxon sets leading from them, respectively (Fig. 1). An NNI on *T* around *e* is obtained by exchanging the subtrees below two nonincident edges from *e*_1_, *e*_2_, *e*_3_, *e*_4_. We denote a new tree by *T_NNI_*.

For each interior edge *e* there are two possible NNIs obtained by exchanging a subtree below *e*_1_ with a subtree below either *e*_3_ or *e*_4_ (note that this is equivalent to swapping the subtree below *e*_2_ with either *e*_4_ or *e*_3_, respectively).

Let us assume that the NNI is applied to edge *e* by swapping *e*_1_ and *e*_3_. The splits corresponding to *e*_1_, *e*_2_, *e*_3_, and *e*_4_ stay unchanged:

\begin{align*}e_1 = A \mid B \cup C \cup D , \\ e_2 = B \mid A
\cup C \cup D , \\ e_3 = C \mid A \cup B \cup D , \\ e_4 = D \mid
A \cup B \cup C.\end{align*}

This also holds true for the edges belonging to subtrees below *e*_1_, *e*_2_, *e*_3_, and *e*_4_ (Fig. 1). Here, if *e*_1_ = *A*|*B* ∪ *C* ∪ *D*, *the subtree below e*_1_ is a subtree with a leaf set *A* and not the union of sets. Hence, the splits corresponding to *e*_1_, *e*_2_, *e*_3_, *e*_4_ and edges below them will be shared by *T* and *T_NNI_*.

The central edge *e* in terms of splits will be changed by the NNI from *A* ∪ *B*|*C* ∪ *D* to *e^NNI^* = *A*∪*D*|*B* ∪ *C*.

It follows from above that *T* and *T_NNI_* are different only in one split; that is,

\begin{align*}\Sigma ( T ) \ \Delta \ \Sigma ( T_{NNI} ) = \{ A
\cup B \mid C \cup D , A \cup D \mid B \cup C \} \end{align*}

and the RF distance between *T* and *T_NNI_* is 2.

We now discuss SPR, a more general topological rearrangement that changes one or more splits of the tree.

**FIG. 2.**
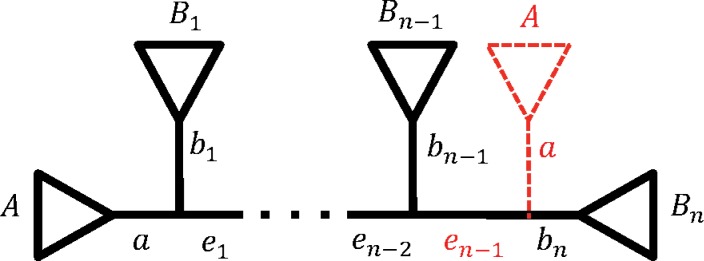
Visualization of SPR. A new tree *T_SPR_* is obtained by pruning the subtree *A* below edge *a* and regrafting it onto edge *b_n_* (dashed red subtree). After SPR is applied, edges *b*_1_ and *e*_1_ are joined and edge *b_n_* is split into *e_n−1_* and *b_n_*. SPR, subtree pruning and regrafting.

An SPR on *T* is represented in Figure 2 (see also Hordijk and Gascuel, 2005). A new tree *T_SPR_* is obtained from *T* by pruning the subtree below edge *a* and regrafting it onto edge *b_n_* (we sometimes refer to such SPR as *n*-SPR). Note, that *n* is at least 3 and if *n* = 3, an SPR is equivalent to an NNI obtained by swapping subtrees belonging to edges *a* and *b*_2_. Let *A*, *B*_1_, …, *B_n_* denote the corresponding taxon sets leading from *a*, *b*_1_, …, *b_n_*, respectively (Fig. 2).

An SPR on *T* changes only the splits of the path edges, namely: for $\forall x \in \{ 1 , \ldots , n - 2 \}$

\begin{align*}e_x = A \cup B_1 \cup \ldots \cup B_x \mid B_{x +
1} \cup \ldots \cup B_n\end{align*}

is changed to

\begin{align*}e_x^{SPR} = B_1 \cup \ldots \cup B_x \mid B_{x + 1}
\cup \ldots \cup B_n \cup A ,\end{align*}

where *e_x_^SPR^* is an edge that corresponds to *e_x_* on a new tree *T_SPR_*. Also, a new edge appears: *e_n_*_− 1_ = *B*_1_ ∪ … ∪ *B_n_*_−1_|*A* ∪ *B_n_*. The rest of splits remain unchanged and are shared by both trees. Hence, for *T* and *T_SPR_* the symmetric difference $\Sigma ( T ) \ \Delta \ \Sigma ( T_{SPR} )$ consists of the following splits:

\begin{align*} & A \cup B_1 \cup \ldots \cup B_x \mid B_{x + 1}
\cup \ldots \cup B_n , \quad \forall x \ \in \ \{ 1 , \ldots , n -
2 \}  , \\ & B_1 \cup \ldots \cup B_x \mid B_{x + 1} \cup \ldots
\cup B_n \cup A , \quad \forall x \ \in \ \{ 2 , \ldots , n - 1 \}
.\end{align*}

The RF distance between *T* and *T_SPR_* is equal to 2 (*n* − 2).

**FIG. 3.**
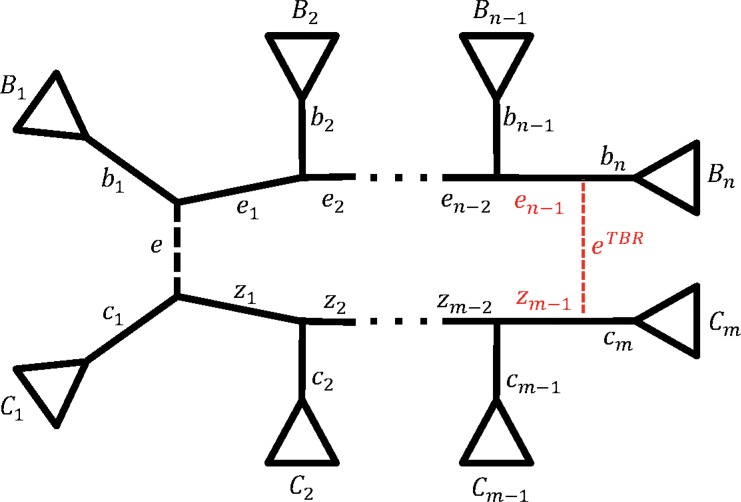
Visualization of TBR. To obtain *T_TBR_*, species tree *T* is cut into two parts (by removing edge *e*), which are further reconnected by joining edges *b_n_* and *c_m_* with *e^TBR^.* Edge *b_n_* is split into *e_n−1_* and *b_n_*, while *c_m_* is split into *z_m−1_* and *c_m_*. Edges *b_1_* and *e_1_* are joined, as well as *c*_1_ and *z*_1_. TBR, tree bisection and reconnection.

The last topological rearrangement we are going to discuss is the TBR. A TBR on *T* is shown in Figure 3, where a new tree *T_TBR_* is obtained from *T* (Fig. 3, in black) by cutting edge *e* and reconnecting edges *b_n_* and *c_m_* with a new edge *e^TBR^* (Fig. 3, red dashed line). Note that *n* or *m* must be greater than 2. W.l.o.g. assume that *m* ≤ *n*. If *n* = 3 and *m* = 2, then a TBR corresponds to an NNI around edge *e*_1_ by swapping subtrees below *e* and *b*_2_. If *n* > 3 and *m* = 2, then a TBR corresponds to an SPR.

TBR only changes the splits corresponding to all path edges (*e_i_* and *z_j_*), but *e*. Namely,

\begin{align*}e = B_1 \cup \ldots \cup B_n \mid C_1 \cup \ldots
\cup C_m = e^{TBR} ,\end{align*}

while for $$\forall x \ \in \ \{  1 , \ldots , n - 2 \} $$

\begin{align*}e_x = C_1 \cup \ldots \cup C_m \cup B_1 \cup \ldots
\cup B_x \mid B_{x + 1} \cup \ldots \cup B_n\end{align*}

is changed to

\begin{align*}e_x^{TBR} = B_1 \cup \ldots \cup B_x \mid B_{x + 1}
\cup \ldots \cup B_n \cup C_1 \cup \ldots \cup C_m\end{align*}

and for $$\forall y \ \in \ \{  1 , \ldots , m - 2 \} $$

\begin{align*}z_y = B_1 \cup \ldots \cup B_n \cup C_1 \cup \ldots
\cup C_y \mid C_{y + 1} \cup \ldots \cup C_m\end{align*}

is changed to

\begin{align*}z_y^{TBR} = C_1 \cup \ldots \cup C_y \mid C_{y + 1}
\cup \ldots \cup C_m \cup B_1 \cup \ldots \cup B_n.\end{align*}

Also two new edges appear

\begin{align*}e_{n - 1} = B_1 \cup \ldots \cup B_{n - 1} \mid B_n
\cup C_1 \cup \ldots \cup C_m , \\ z_{m - 1} = C_1 \cup \ldots
\cup C_{m - 1} \mid C_m \cup B_1 \cup \ldots \cup B_n.\end{align*}

The remaining splits stay unchanged. Hence, for *T* and *T_TBR_* the symmetric difference $\Sigma ( T ) \ \Delta \ \Sigma ( T_{TBR} )$ is a set consisting of the following splits

\begin{align*}C_1 \cup \ldots \cup C_m \cup B_1 \cup \ldots \cup
B_x \mid B_{x + 1} \cup \ldots \cup B_n , \quad \forall x \in \{ 1
, \ldots , n - 2 \}  , \\ B_1 \cup \ldots \cup B_x \mid B_{x + 1}
\cup \ldots \cup B_n \cup C_1 \cup \ldots \cup C_m , \quad \forall
x \in \{ 2 , \ldots , n - 1 \}  , \\ B_1 \cup \ldots \cup B_n \cup
C_1 \cup \ldots \cup C_y \mid C_{y + 1} \cup \ldots \cup C_m ,
\quad \forall y \in \{ 1 , \ldots , m - 2 \}  , \\ C_1 \cup \ldots
\cup C_y \mid C_{y + 1} \cup \ldots \cup C_m \cup B_1 \cup \ldots
\cup B_n , \quad \forall y \in \{ 2 , \ldots , m - 1 \}
.\end{align*}

Therefore, the RF distance between *T* and *T_TBR_* is 2 (*n* + *m* − 4).

## 3. Consequences of Topological Rearrangements Applied to a Species Tree

In the following we discuss how the topological rearrangement of the species tree *T* influences the topology of the partition trees and start with the simplest operation, an NNI.

**Proposition 1.** *Let *e* be an interior edge and *e*_1_, *e*_2_, *e*_3_, *e*_4_ the four edges adjacent to *e* with *A*, *B*, *C*, *D* being the taxon sets leading from the corresponding edges (Fig. 1). Let a new tree *T_NNI_* be obtained from *T* via NNI. For a partition with a taxon set *Y*, the topologies of *T*|*Y* and *T_NNI_*|*Y* are different iff *Y* has at least one representative taxon in each subset *A*, *B*, *C*, *D*.*

**Proof.**

W.l.o.g. assume that *T_NNI_* is obtained from *T* via swapping of subtrees below *e*_1_ and *e*_3_. Then $$\Sigma ( T ) \ \Delta \ \Sigma \left( T_{NNI} \right) = \{ A
\cup B \mid C \cup D , \ A \cup D \mid B \cup C \} $$ and as a consequence for corresponding partition trees we have

\begin{align*}\Sigma ( T \mid Y ) \ \Delta \ \Sigma ( T_{NNI}
\mid Y ) = \{  ( A \cup B ) \cap Y \mid ( C \cup D ) \cap Y , ( A
\cup D ) \cap Y \mid ( B \cup C ) \cap Y \} .\end{align*}

**FIG. 4.**
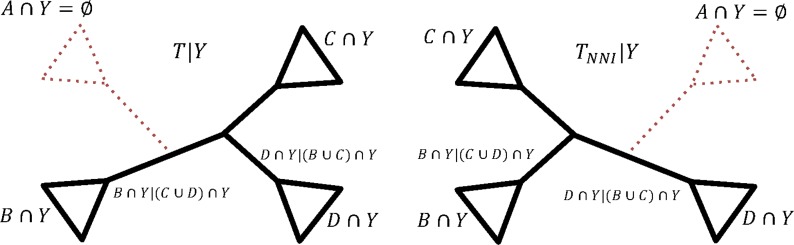
An example when an NNI on *T* does not change the topology of *T|Y.* Solid lines correspond to two induced partition trees before (*T|Y*) and after (*T_NNI_|Y*) the NNI was applied to edge *e* on *T* by swapping the subtrees below *e*_1_ and *e*_3_ (Fig. 1). In this case, *Y* does not have a representative in *A* (i.e., *A* ∩ *Y* = ∅); therefore, (*A* ∪ *B*)∩*Y|(C∪D)∩Y* =*B∩Y|(C∪D)∩Y* and *(A∪D)∩Y|(B∪C)∩Y = D∩Y|(B∪C)∩Y*. Since the splits *B∩Y|(C∪D)∩Y* and *D∩Y|(B∪C)∩Y* are shared by *T|Y* and *T_NNI_|Y*, then *Σ(T|Y)* Δ *Σ(T_NNI_|Y)* = ∅ and RF distance between *T|Y* and *T_NNI_|Y* is 0.

It is easy to show that if at least one set from *A* ∩ *Y*, *B* ∩ *Y*, *C* ∩ *Y*, *D* ∩ *Y* were empty, then both splits (*A* ∪ *B*) ∩ *Y*|(*C* ∪ *D*) ∩ *Y* and *(A ∪ D) ∩ Y|(B ∪ C) ∩ Y* coincide with splits shared by *T*|*Y* and *T_NNI_*|*Y* (e.g., see Fig. 4). Hence, $$\Sigma ( T \mid Y ) \ \Delta \ \Sigma \left( T_{NNI} \mid Y
\right) = \emptyset$$ and the RF distance between these trees would be 0. Therefore, for *T*|*Y* and *T_NNI_*|*Y* to have different topologies, all *A* ∩ *Y*, *B* ∩ *Y*, *C* ∩ *Y*, *D* ∩ *Y* must be nonempty, meaning that *Y* has to have at least one representative in each subset *A*, *B*, *C*, *D*.   ■

In simple words, if some intersections of *A*, *B*, *C*, *D* with *Y* are empty, then a partition tree does not have a corresponding quartet structure for the NNI to be applied to and edge *e* loses its centrality or interior feature (see, e.g., Fig. 4). When this happens, the topology of the partition tree *T*|*Y* is not affected by the NNI applied to *e* on the species tree *T*.

We next specify the condition when an SPR changes the topology of partition tree.

**Proposition 2.** *Let tree *T* be in the form shown in Figure 2, and a new tree *T_SPR_* is obtained with SPR by pruning subtree below edge *a* and regrafting it onto *b_n_*.*

Then for a partition with a taxon set *Y* the following is true:

(i) the topologies of *T*|*Y* and *T_SPR_*|*Y* are different, if *Y* has at least one representative in *A* and in at least another three subsets from *B*_1_, *B*_2_, …, *B_n_*;

(ii) this SPR will correspond to an SPR on *T*|*Y* obtained by pruning the subtree below edge with a split *A* ∩ *Y*|(*B*_1_ ∪ … ∪ *B_n_*) ∩ *Y* and regrafting it onto edge with split $$B_k \cap Y \mid \left( \cup_{i \in \{ 1 , \ldots , n \}
\setminus  k} \ B_i \cup A \right) \cap Y$$, where $$k = \max \nolimits_{1 \le i \le n} \{ i \mid B_i \cap Y \ne \emptyset \} $$.

**Proof.**

(i) The symmetric difference $\Sigma ( T ) \ \Delta \ \Sigma ( T_{SPR} )$ consists of the following splits

\begin{align*}A \cup B_1 \cup \ldots \cup B_x \mid B_{x + 1} \cup
\ldots \cup B_n , \quad \forall x \ \in \ \{ 1 , \ldots , n - 2 \}
, \\ B_1 \cup \ldots \cup B_x \mid B_{x + 1} \cup \ldots \cup B_n
\cup A , \quad \forall x \ \in \ \{ 2 , \ldots , n - 1 \}
.\end{align*}

As a consequence for the induced partition trees *T*|*Y* and *T_SPR_*|*Y*, the symmetric difference of *Σ*(*T*|*Y*) and *Σ*(*T_SPR_*|*Y*) consists of

\begin{align*} \left( A \cup B_1 \cup \ldots \cup B_x \right)
\cap Y \mid \left( B_{x + 1} \cup \ldots \cup B_n \right) \cap Y ,
\quad \forall x \ \in \ \{ 1 , \ldots , n - 2 \}  , \\ \left( B_1
\cup \ldots \cup B_x \right) \cap Y \mid \left( B_{x + 1} \cup
\ldots \cup B_n \cup A \right) \cap Y , \quad \forall x \ \in \ \{
2 , \ldots , n - 1 \}.\end{align*}

It is easy to see that if *A* ∩ *Y* = ∅, then all these splits would be shared by both partition trees, that is, *Σ*(*T*|*Y*) Δ *Σ*(*T_SPR_*|*Y*) = ∅ and the RF distance between *T*|*Y* and *T_SPR_*|*Y* would be 0. Therefore, *Y* must have at least one representative in *A*.

**FIG. 5.**
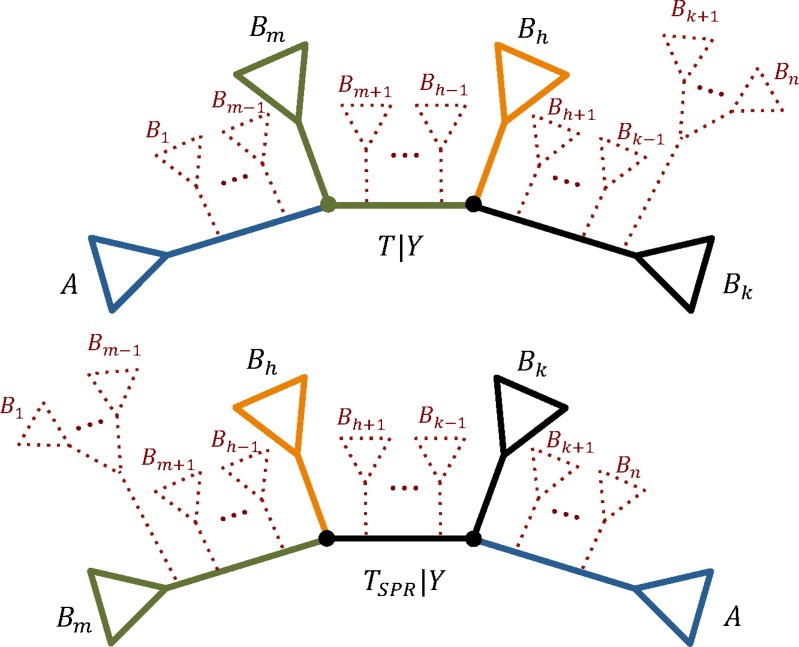
An example when *n*-SPR on *T* is a 3-SPR (or NNI) on *T|Y*. There are two induced partition trees (solid lines): before (*T|Y*) and after (*T_SPR_|Y*) an SPR was applied on *T* by pruning the subtree below edge *a* and regrafting it onto *b_n_* (Fig. 2). The three dots denote all the subtrees between the corresponding pair of subtrees on the species trees *T* and *T_SPR_*. Here, only *A, B_m_, B_h_,* and *B_k_* have at least one representative in *Y* and ∀*j*∈*{*1, *… , n}\{m, h, k}*: *B_j_* have no taxa in common with *Y*.

For *T*|*Y* and *T_SPR_*|*Y* to have different topologies, an SPR on *T* should correspond to at least an NNI on *T*|*Y*. Hence, *T*|*Y* must have a corresponding quartet structure and together with *A* at least another three subsets from *B*_1_, *B*_2_, …, *B_n_* should have at least one representative in *Y*. W.l.o.g. assume that together with *A* also *B_m_*, *B_h_*, *B_k_* (1 ≤ *m* < *h* < *k* ≤ *n*) have at least one representative in *Y* while $$B_j \cap Y = \emptyset \ \forall j \ \in \{ 1 , \ldots , n \}
\setminus \{ m , h , k \} $$ (see, e.g., Fig. 5). Then

\begin{align*}\Sigma ( T \mid Y ) \ \Delta \ \Sigma \left(
T_{SPR} \mid Y \right) = \{  \left( A \cup B_m \right) \cap Y \mid
\left( B_h \cup B_k \right) \cap Y , \ \left( B_m \cup B_h \right)
\cap Y \mid \left( B_k \cup A \right) \cap Y \} .\end{align*}

Thus, the RF distance between *T*|*Y* and *T_SPR_*|*Y* is 2.

(ii) Let *I* = {*i*_1_,., *i*_k_} be the set of all indices, such that $\forall i \in I: \ B_i \cap Y \ne \emptyset$ and let $1 \le i_1 < \cdots < i_k \le n$.

For edge *a* = *A*|*B*_1_ ∪ … ∪ *B_n_* its corresponding split on the partition tree *T*|*Y* is equal to

\begin{align*}A \cap Y \mid \left( B_1 \cup \ldots \cup B_n
\right) \cap Y = A \cap Y \mid \cup_{i \in I} \left( B_i \cap Y
\right) .\end{align*}

Similarly for $e_{{i}_{k}{ - 1}}$ its corresponding split on *T*|*Y*

\begin{align*}\left( A \cup B_1 \cup \ldots \cup B_{{i}_{{k}}{ -
1}} \right) \cap Y \mid { \left( B_{i_k} \cup \ldots \cup B_n
\right) \cap Y = \left( \cup_{i \in I \setminus {i_k}} B_i \cup A
\right) \cap Y} \mid B_{i_k} \cap Y ,\end{align*}

and for $e_{{i}_{{k}}{ - 1}}^{SPR}$ its corresponding split on the partition tree *T_SPR_*|*Y*

\begin{align*}\left( B_1 \cup \ldots \cup B_{{i}_{{k}}{ - 1}}
\right) \cap Y \mid \left( B_{{i}_{k}} \cup \ldots \cup B_n \cup A
\right) \cap Y = \left( \cup_{i \in I \setminus {i_k}} B_i \right)
\cap Y \mid \left( B_{i_k} \cup A \right) \cap Y.\end{align*}

The above means that an edge on *T*|*Y* with split $$\left( \cup _{i \in I \setminus {i_k}} B_i \cup A \right) \cap
Y \mid B_{i_k} \cap Y$$ was divided by an edge with split $$A \cap Y \mid \cup_{i \in I} \left( B_i \cap Y \right)$$ in two edges (see also Fig. 5, where *I* = {*i*_1_, *i*_2_, *i*_3_}). Therefore, regrafting onto edge *b_n_* on *T* corresponds to regrafting onto edge with a split $$B_{i_k} \cap Y \mid \left( { \cup_{{i \in \{ 1 , . , n \}
\setminus i_k}}} B_i \cup A \right) \cap Y$$ on partition tree *T*|*Y*. And since $$1 \le i_1 < \cdots < i_k \le n$$, then $$i_k = \max \nolimits_{1 \le i \le n} \{ i \mid B_i \cap Y \ne \emptyset \} $$.   ■

In other words, Proposition 2 states that an SPR on *T* changes the topology of *T*|*Y* if the structure of *T* from Figure 2 corresponds to at least a quartet structure on *T*|*Y* (e.g., Fig. 5). In this case, *n*-SPR on *T* is a 3-SPR (or NNI) on *T*|*Y*.

We now discuss TBR and the topological change of a partition tree as a consequence of TBR on species tree.

**Proposition 3.** *Let tree *T* be in the form shown in Figure 3 and a new tree *T_TBR_* is obtained by cutting edge *e* and reconnecting *b_n_* and *c_m_* with a new edge.*

Then for a partition with a taxon set *Y* the following is true:

(i) the topologies of *T*|*Y* and *T_TBR_*|*Y* are different if either of the following conditions is satisfied:

• *Y* has at least one representative in at least one subset from *B*_1_, *B*_2_, … , *B_n_* and in at least another three subsets from *C*_1_, *C*_2_, … ,*C_m_*
• *Y* has at least one representative in at least one subset from *C*_1_, *C*_2_, … , *C_m_* and in at least another three subsets from *B*_1_, *B*_2_, … , *B_n_*

(ii) this TBR will correspond to a TBR on *T*|*Y* obtained by cutting the edge with split (*B*_1_ ∪ … ∪ *B_n_*) ∩*Y*|(*C*_1_ ∪ … ∪ *C_m_*) ∩ *Y* and reconnecting edges with splits *B_k_* ∩ *Y*|(∪_*i*ε{1,.,*n*}\*k*_ *B_i_* ∪ *C*_1_ ∪ … ∪ *C_m_*) ∩ *Y* and *C_h_* ∩ *Y*|(∪_*j*ε{1,.,*m*}\*h*_ *C*_*j*_ ∪ *B*_1_ ∪ … ∪ *B_n_*) ∩ *Y*, where *k* = max_1≤*i*≤n_ {*i* | *B_i_* ∩ *Y* ≠ ∅} and *h* = max_1≤*j*≤*m*_ {*j* | *C_j_* ∩ *Y* ≠ ∅}.

**Proof.**

(i) The symmetric difference *Σ* (*T*) Δ *Σ* (*T_TBR_*) consists of the following splits:

\begin{align*}C_1 \ \cup \ldots \cup \ C_m \cup B_1 \cup \ldots
\cup B_x \mid B_{x + 1} \cup \ldots \cup B_n , \quad \forall x \in
\{ 1 , \ldots , n - 2 \}  , \\ B_1 \ \cup \ldots \cup \ B_x \mid
B_{x + 1} \cup \ldots \cup B_n \cup C_1 \cup \ldots \cup C_m ,
\quad \forall x \in \{ 2 , \ldots , n - 1 \}  , \\ B_1 \ \cup
\ldots \cup \ B_n \cup C_1 \cup \ldots \cup C_y \mid C_{y + 1}
\cup \ldots \cup C_m , \quad \forall y \in \{ 1 , \ldots , m - 2
\}  , \\ C_1 \ \cup \ldots \cup \ C_y \mid C_{y + 1} \cup \ldots
\cup C_m \cup B_1 \cup \ldots \cup B_n , \quad \forall y \in \{ 2
, \ldots , m - 1 \}  , \end{align*}

As a consequence, the symmetric difference $\Sigma ( T \mid Y ) \Delta \Sigma ( T_{TBR} \mid Y )$ consists of

\begin{align*} ( C_1 \cup \ldots \cup C_m \cup B_1 \cup \ldots
\cup B_x ) \cap Y \mid ( B_{x + 1} \cup \ldots \cup B_n ) \cap Y ,
\quad \forall x \in \{ 1 , \ldots , n - 2 \}  , \\ ( B_1 \cup
\ldots \cup B_x ) \cap Y \mid ( B_{x + 1} \cup \ldots \cup B_n
\cup C_1 \cup \ldots \cup C_m ) \cap Y , \quad \forall x \in \{ 2
, \ldots , n - 1 \}  , \\ ( B_1 \cup \ldots \cup B_n \cup C_1 \cup
\ldots \cup C_y ) \cap Y \mid ( C_{y + 1} \cup \ldots \cup C_m )
\cap Y , \quad \forall y \in \{ 1 , \ldots , m - 2 \}  , \\ ( C_1
\cup \ldots \cup C_y ) \cap Y \mid ( C_{y + 1} \cup \ldots \cup
C_m \cup B_1 \cup \ldots \cup B_n ) \cap Y , \quad \forall y \in
\{ 2 , \ldots , m - 1 \}  , \end{align*}

It is easy to see that if ∀*i* ε {1,.,*n*}: *B_i_ ∩ Y* = ∅, then all these splits would be shared by both partition trees; that is *Σ*(*T*|*Y*) Δ *Σ*(*T_TBR_*|*Y*) = ∅ and the RF distance between *T*|*Y* and *T_TBR_*|*Y* would be 0. Therefore, *Y* must have at least one representative in at least one from *B*_1_, *B*_2_, …, *B_n_*. Similarly, *Y* must have at least one representative in at least one from *C*_1_, *C*_2_, … , *C_m_*.

W.l.o.g. assume that *B_k_* ∩ *Y* ≠ ∅ and *C_h_* ∩ *Y* ≠ ∅, where 1 ≤ *k* ≤ *n* and 1 ≤ *h* ≤ *m*.

Partition trees *T*|*Y* and *T_TBR_*|*Y* will have different topologies if a TBR on *T* corresponds to at least an NNI on *T*|*Y*. Hence, the partition tree *T*|*Y* must have a corresponding quartet structure and together with *B_k_* and *C_h_* at least other two subsets from the remaining *B_i_* and *C_j_* should have at least one representative in *Y*.

**FIG. 6.**
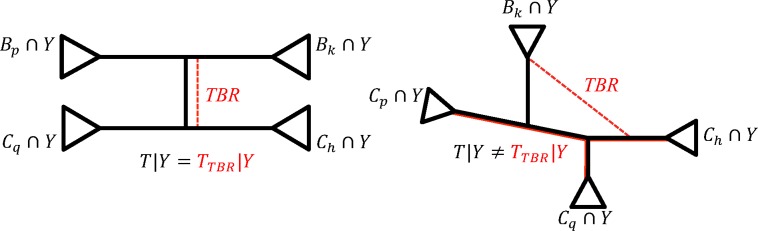
Examples of corresponding TBRs on partition trees. Two partition trees with topologies before (*T|Y*, in black) and after (*T_TBR_|Y*, in red) the TBR were applied to the species tree. For simplicity we do not show the pruned subtrees for which *B_i_*∩*Y* = *∅* and *C*_*j*_∩*Y* = *∅.* On the left is an example case when the topology of partition tree remains unchanged after TBR. On the right is the simplest case when the TBR changes the topology of partition tree. In this case a TBR on species tree corresponds to an NNI on partition tree.

W.l.o.g. assume that together with *B_k_* and *C_h_* also *C_p_*, *C_q_* (1 ≤ *p* < *q* < *h* ≤ *m*) have at least one representative in *Y* (Fig. 6, right panel). Then it is easy to show that

\begin{align*}\Sigma ( T \mid Y ) \Delta \Sigma ( T_{TBR} \mid Y
) = \{  ( B_k \cup C_p ) \cap Y \mid ( C_q \cup C_h ) \cap Y , (
C_p \cup C_q ) \cap Y \mid ( C_h \cup B_k ) \cap Y \} \end{align*}

and RF distance between *T*|*Y* and *T_TBR_*|*Y* is 2.

Similarly, one can show that if together with *B_k_* and *C_h_* also *B_p_*, *B_q_* (1 ≤ *p* < *q* < *k* ≤ *n*) have at least one representative in *Y*, then RF distance between *T*|*Y* and *T_TBR_*|*Y* is also 2.

In contrast, if *Y* has at least one representative in *B_k_*, *C_h_* and also in *B_p_*, *C_q_* (1 ≤ *p* < *k* ≤ *n* and 1 ≤ *q* < *h* ≤ *m*), then $$\Sigma ( T \mid Y ) \Delta \Sigma ( T_{TBR} \mid Y ) =
\emptyset$$ and RF distance is 0 (Fig. 6, left panel).

(ii) Let *I* = {*i*_1_,.,*i_k_*} be the set of all indices such that $\forall i \in I: B_i \cap Y \neq \emptyset$ and let 1 ≤ *i*_1_ < … < *i_k_* ≤ *n*. Similarly, let *J* = {*j*_1_,.,*j_h_*} be the set of all indices such that $\forall j \in J: C_j \cap Y \neq \emptyset$ and let 1 ≤ *j*_1_ < … < *j_h_* ≤ *m*. Then for edge

\begin{align*}e = B_1 \cup \ldots \cup B_n \mid C_1 \cup \ldots
\cup C_m\end{align*}

the corresponding split on *T*|*Y* is

\begin{align*}\cup _{i \in I} B_i \cap Y \mid \cup_{j \in J} C_j
\cap Y.\end{align*}

For edge

\begin{align*}e_{i_k{-1}} = C_1 \cup \ldots \cup C_m \cup B_1
\cup \ldots \cup B_{{i_k} - 1} \mid B_{{i_k} } \cup \ldots \cup
B_n\end{align*}

its corresponding split on tree *T*|*Y* is

\begin{align*} \left( C_1 \cup \ldots \cup C_m \cup B_1 \cup
\ldots \cup B_{{i_k} - 1} \right) \cap Y \mid \left( B_{{i_k} }
\cup \ldots \cup B_n \right) \cap Y = \\ = \left( \cup _{j \in J}
C_j \cap Y \right) \cup \left( \cup _{i \in I \setminus i_k} B_i
\cap Y \right) \mid B_{i_k} \cap Y.\end{align*}

Similarly, for the corresponding edge on $$T_{TBR} \ e_{{i_k}{-1}}^{TBR} = B_1 \cup \ldots \cup B_{{i_k} -
1} \mid B_{{i_k} } \cup \ldots \cup B_n \cup C_1 \cup \ldots \cup
C_m$$ its split on *T_TBR_*|*Y* is

\begin{align*} \left( B_1 \cup \ldots \cup B_{{i_k} - 1} \right)
\cap Y \mid \left( B_{{i_k} } \cup \ldots \cup B_n \cup C_1 \cup
\ldots \cup C_m \right) \cap Y = \\ = \left( \cup _{i \in I
\setminus {i_k}} B_i \cap Y \right) \mid \left( B_{{i_k} \cap Y}
\right) \cup \left( \cup _{j \in J} C_j \cap Y \right)
.\end{align*}

For edges

\begin{align*}z_{{j_h}-1} = {B_1} \cup \ldots \cup B_n \cup C_1
\cup \ldots \cup C_{j_{h} - 1} \mid C_{{j_h}} \cup \ldots \cup
C_m\end{align*}

and

\begin{align*}z_{{j_h}-1}^{TBR} = C_1 \cup \ldots \cup C_{j_{h}
{-1}} \mid C_{{j_h}} \cup \ldots \cup C_m \cup B_1 \cup \ldots
\cup B_n\end{align*}

their corresponding splits on *T*|*Y* and *T_TBR_*|*Y* are

\begin{align*}\left( \cup _{i \in I} B_i \cap Y \right) \cup
\left( \cup _{j \in J \setminus {j_h}} C_j \cap Y \right) \mid
C_{{j_h}} \cap Y\end{align*}

and

\begin{align*}\left( \cup _{j \in J \setminus {j_h}} C_j \cap Y
\right) \mid \left( C_{{j_h}} \cap Y \right) \cap \left( \cup _{i
\in I} B_i \cap Y \right)\end{align*}

respectively. The above means that edges on *T*|*Y* with corresponding splits

\begin{align*}\left( \cup _{j \in J} \ C_j \cap Y \right) \cup
\left( \cup _{i \in I \setminus i_k} B_i \cap Y \right) \mid
B_{{i_k}} \cap Y\end{align*}

and

\begin{align*}\left( \cup _{i \in I} \ B_i \cap Y \right) \cup
\left( \cup _{j \in J \setminus {j_h}} C_j \cap Y \right) \mid
C_{j_h} \cap Y\end{align*}

were reconnected on $$T_{TBR} \mid Y \ { \rm by} \cup_{i \in I} B_i \cap Y \mid
\cup_{j \in J} C_j \cap Y$$. Since 1 ≤ *i*_1_ < … < *i_k_* ≤ *n* and 1 ≤ *j*_1_ < … < *j_h_* ≤ *m*, then *i_k_* = max_1≤*i*≤*n*_{*i* | *B*_*i*_ ∩ *Y* ≠ ∅} and *j_h_* = max _1≤*j*≤*m*_{*j* | *C_j_* ∩ *Y* ≠ ∅}.   ■

## 4. Partial Terraces

### 4.1. Definition of partial terraces

In this section we discuss *partial terraces* that generalize the terrace concept (Sanderson et al., 2011), which we call *full terrace* for clarity. When comparing the two trees in a partitioned framework, we compare the sets of their induced partition trees. If the sets are identical, then the two trees belong to one full terrace. Sanderson et al. (2011) showed that the number of trees on one full terrace can be quite large. Large full terraces pose a problem in phylogenetic inference, since they may abort tree search prematurely or even if an optimal tree has been found, this tree is by no means unique. To reduce this problem, it is possible to reduce the terrace size by, for example, choosing a different partition scheme (Sanderson et al., 2015) or by excluding some taxa from the analysis.

Now, if two species trees *T*_1_ and *T*_2_ share only a subset of identical induced partition trees, then we say that they belong to the same *partial terrace*. The log-likelihoods and parsimony scores of identical partition trees *T*_1_|*Y_i_* and *T*_2_|*Y_i_* are the same. Obviously, partial terraces occur more frequently than full terraces (see below). Large partial terraces can be still problematic for tree search algorithms. On the other hand, partial terraces provide the potential to reduce computation time.

### 4.2. Occurrence of partial terraces in real data

In this section we evaluate how often partial terraces occur in real alignments. By no means do we intend to make a full exploration of potential computing time that may be saved since the performance of the particular software will depend on the data structures and particular implementation used for the tree space exploration.

To elucidate the occurrence of partial terraces and full terraces, we analyzed seven recently published alignments (Table 1). Alignments have different numbers of taxa ranging from 69 to 404 taxa. The number of partitions (here, genes) varies from 11 to 79.

**Table 1.** Alignments Used to Study the Occurrence of Partial Terraces During the Tree Search

*Type and ID*	*No. of species*	*No. of genes*	*Missing data (%)*	*Source*
DNA1	128	32	30	Stamatakis and Alachiotis (2010)
DNA2	237	74	72	Nyakatura and Bininda-Emonds (2012)
DNA3	372	79	66	Springer et al. (2012)
DNA4	404	11	60	Stamatakis and Alachiotis (2010)
AA1	69	31	35	De Queiroz et al. (1995)
AA2	70	35	34	
AA3	72	51	35	

For each alignment we performed a maximum likelihood tree search using IQ-TREE (Nguyen et al., 2015) under the edge-unlinked (EUL) partition model assuming a GTR+Γ (Lanave et al., 1984; Yang, 1994) model for all partitions. We collected all the intermediate trees encountered during the search. For each intermediate tree *T*, we explored all trees *T_NNI_* in its NNI neighborhood. We examined partial terraces of each *T_NNI_* and *T* by computing how many induced partition trees are shared between them.

Apart from intermediate trees collected during the tree search, we also analyzed NNI neighborhoods for 1000 random Yule–Harding (YH) trees (Harding, 1971) for each tested alignment.

**FIG. 7.**
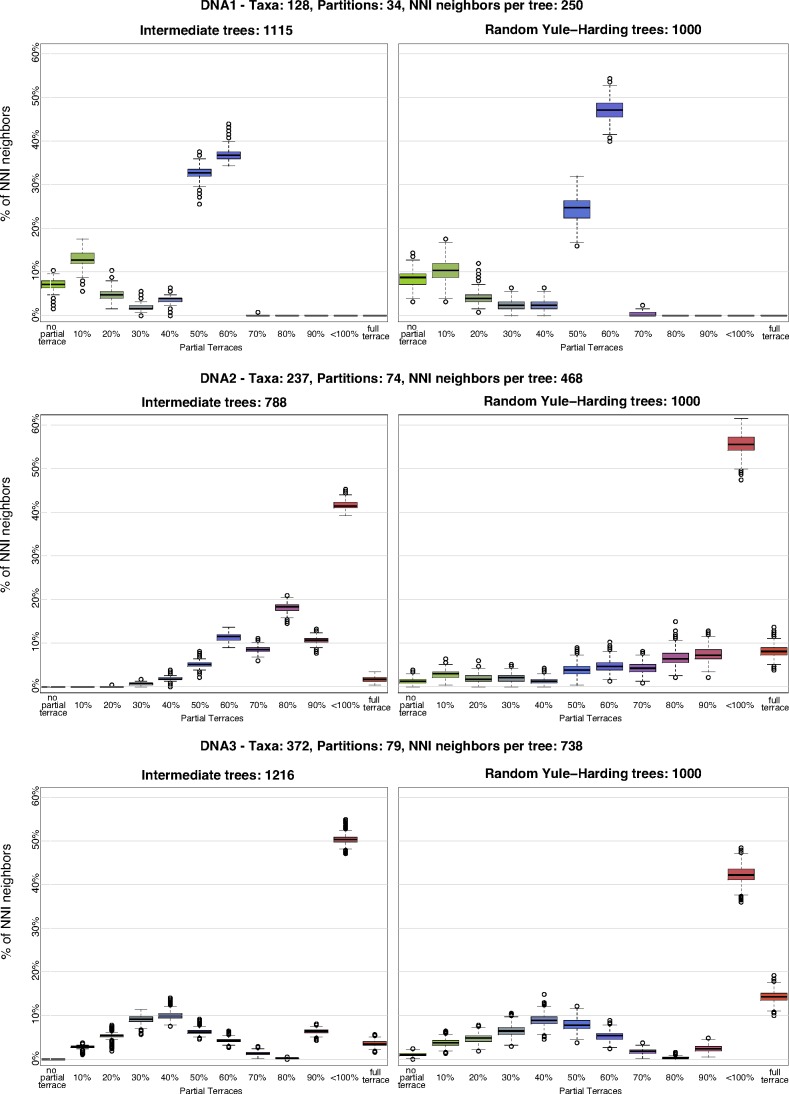
NNI neighborhood analysis for alignments DNA1 (top), DNA2 (middle), and DNA3 (bottom).

We defined 12 bins based on the percentage of shared induced partition trees between *T* and *T_NNI_* (Table 2) and counted how many *T_NNI_* trees fall into each bin. Table 3 shows the mean percentage of *T_NNI_* trees that fall into the corresponding bin for the intermediate trees. Figure 7 displays the boxplots for the first three alignments from Table 1 either for the IQ-TREE search trees (left column) or the random YH trees (right column) (see Supplementary Figs. S1–S4 for the remaining alignments; Supplementary Material is available online at www.liebertonline.com/cmb).

**Table 2.** Partial Terrace Bins Based on the Percentage of the Shared Partition Trees Between *T* and *T_NNI_*

*Name*	*Percentage of shared partition trees out of the total number of partition trees*
No partial terrace (PT)	= 0%, the topologies of all partition trees are pairwise different between *T* and *T_NNI_*
PT1	(0%,10%]
PT2	(10%, 20%]
PT3	(20%, 30%]
…	…
PT9	(80%, 90%]
PT10	(90%, 100%)
Full terrace	= 100%, *T* and *T_NNI_* belong to one terrace

**Table 3.** Mean Percentage of Trees from NNI Neighborhood of Intermediate Trees Falling into Corresponding Partial Terrace Bin

	*No PT (%)*	*PT1 (%)*	*PT2 (%)*	*PT3 (%)*	*PT4 (%)*	*PT5 (%)*	*PT6 (%)*	*PT7 (%)*	*PT8 (%)*	*PT9 (%)*	*PT10 (%)*	*Full terrace (%)*
DNA1	7.14	12.97	4.85	1.80	3.55	32.59	37.11	0	0	0	0	0
DNA2	0	0	0.02	0.63	1.82	5.07	11.31	8.69	18.19	10.77	41.75	1.75
DNA3	0	2.75	5.38	9.22	10.06	6.32	4.34	1.33	0.23	6.38	50.36	3.63
DNA4	0.35	0.26	1.88	4.06	5.20	6.56	8.77	11.34	16.48	23.37	17.68	4.05
AA1	12.11	10.64	7.47	8.10	6.35	15.42	11.28	8.20	10.50	7.18	2.76	0
AA2	8.73	11.90	6.77	9.10	11.22	9.08	16.27	10.95	11.63	2.92	1.44	0
AA3	12.25	11.62	4.07	7.15	3.04	15.47	15.43	10.40	7.55	7.92	4.85	0.26

Intermediate and random trees have similar percentages of *T_NNI_* trees across different bins (Fig. 7 and Supplementary Figs. S1–S4). This suggests that the general picture of partial terraces is mainly determined by the spread of missing data in the supermatrix and is less dependent on the actual tree topology. Moreover, increasing the number of taxa tends to decrease the variance of *T_NNI_* percentage within each bin (for both intermediate and random trees).

**FIG. 8.**
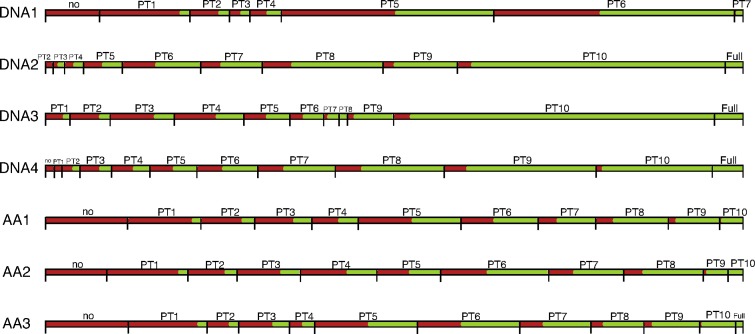
Visualization of NNI neighborhoods and potential computational savings. Each horizontal line reflects the NNI neighborhood for each test alignment, that is 100% of *T_NNI_* trees. These neighborhoods are divided into partial terrace bins (Table 2) and depicted here by horizontal segments. The length of each segment corresponds to the mean percentage of *T_NNI_* trees falling into the bin (Table 3). Each segment is composed of a green and a red bar, corresponding to the fractions of partition trees that are shared and not shared between *T* and *T_NNI_*, respectively. Basically, green bars indicate potential computational savings when accounting for partial and full terraces during the trees search.

Figure 8 integrates the information from Tables 2 and 3 and provides rough estimates of potential computational savings if accounting for partial and full terraces. The green bars reflect the average percentage of identical induced partition trees when *T_NNI_* is compared to *T*. For example, for DNA1 there is no full terrace, but we observe partial terraces that may lead to a reduction of about 38% (the percentage of green bars) in computation time.

There is a full terrace for DNA2, but it consists of only 1.75% of the NNI neighborhood, whereas partial terraces constitute the remaining 98.25% and lead to a potential reduction of computations of about 80% (the percentage of green bars). In fact, since no *T_NNI_* tree falls into “no PT” bin, we can save some computation time for all the trees encountered during tree search. Similar trend is observed for DNA3 and DNA4 with the predicted timesaving of 71% for each alignment.

## 5. Advantages of Using Induced Partition Trees in Maximum Likelihood Inference

In maximum likelihood inference, after applying a topological rearrangement on *T*, one needs to optimize the edge lengths of a new tree *T_NEW_*. Therefore, together with the topological changes of partition trees, it is important to consider how topological rearrangement on *T* influences edge length optimization.

In the following we discuss two partition models commonly used in likelihood inferences, EUL and edge-linked (EL), and the advantages of using induced partition trees for either model (Yang, 1996).

We start by considering the most general partition model, EUL. Given a species tree *T*, we first obtain the corresponding induced partition trees. Under the EUL model, the edge lengths of the partition trees are optimized separately. The edge lengths of *T* are then computed from the corresponding edges lengths inferred on the partition trees, for example, as mean edge length.

Therefore, if the topological rearrangement on *T* does not change the topology of a partition tree *T*|*Y*, no edge length optimization is necessary and, as a result, the optimal partition tree likelihood remains unchanged after such a topological rearrangement on *T*. Let *T_NEW_* be a tree obtained from *T* by some topological rearrangement.

Under EUL partition model there is no need to optimize the edge lengths of partitioned trees shared between T and T_NEW_. As a result, the log-likelihood of the corresponding partition trees is the same.

In contrast to the EUL model, the edges between *T* and partition trees are linked in the EL model. That means that there is only one set of edge lengths for *T* and partition trees with the possibility of rescaling edge lengths of each partition tree by a partition-specific evolutionary rate. Therefore, the optimization of edge lengths is done on the species tree. Even if a topological rearrangement on *T* does not change the topology of partition tree, it still affects the optimal partition tree likelihood via optimization of edge lengths. This is also the reason why full terraces cannot occur under the EL model (Sanderson et al., 2015). Theoretically, one would need to optimize each edge on the species tree, which would definitely influence the partition tree edge lengths and also the likelihood. But in practice, to save computations, one only optimizes those edges in the vicinity of topological changes (Stamatakis et al., 2005; Guindon et al., 2010; Nguyen et al., 2015). For example, for an NNI, one reoptimizes only five edge lengths (*e*, *e*_1_, *e*_2_, *e*_3_, *e*_4_) around the swap. Under the EL model, such a particular feature of practical optimization can take an advantage when considering the induced partition trees.

*Given a partition tree with taxon set Y and an edge e on T with the corresponding split A*|*B, if A* ∩ *Y* = ∅ *or B* ∩ *Y* = ∅ *then the optimization of e does not affect the likelihood of T*|*Y.*

In this case, a split *A*|*B* does not have a corresponding split in *Σ*(*T*|*Y*), and therefore edge *e* is not linked to any edge on *T*|*Y*. This observation can be exploited to save computing time.

## 6. Discussion

We have shown that it is advantageous to identify and account for full and partial terraces during the tree search in phylogenomics. One main advantage is the saving of computation time. If two trees belong to the same full or partial terrace, then one needs to compute the objective function for the identical partition trees only once. The values of objective function will be the same for these partition trees. The larger the number of identical partition trees between species trees, the more computation time can be saved.

From the conditions discussed in the previous sections, the topological rearrangement that benefits the most from partial terraces is obviously NNI. It is intuitive that NNI applied to the species tree will not change the topology of partition trees more often than SPR or TBR. However, in tree searches one typically applies short SPR (e.g., RAxML); that is, the number of edges between the pruning and the regrafting edges are much smaller than the number of taxa. The same is true for TBR. And since one also expects short SPR and short TBR to result in no change of partition trees quite often for sparse supermatrices, partial terraces are also beneficial for these rearrangements.

Moreover, the use of induced partition trees has another advantage that long SPR or TBR on a species tree *T*, as a result of missing data, might correspond to a much shorter SPR or TBR on *T*|*Y*. This leads to computation saving even if SPR or TBR changes the topology of the induced partition trees.

Here, we elucidated the frequent existence of partial terraces in practice via NNI neighborhoods, showing that partial terraces are not only a theoretical concept, but also have practical implications in phylogenomics. The predicted timesaving for the examined real alignments is only the rough estimate, since we treated the alignment lengths per partition as equal. If the length of alignment corresponding to the shared partition trees is relatively large compared to the whole supermatrix, then one expects even more speed up.

Another important factor for timesaving is the actual implementation of search strategies in the particular software. We plan to implement efficient techniques to take full advantage of partial and full terraces in IQ-TREE. A more thorough analysis of such techniques will be presented elsewhere.

## Supplementary Material

Supplemental data
