# Supplementary material for: Consequences of Common Topological Rearrangements for Partition Trees in Phylogenomic Inference
Source: J Comput Biol. 2015 Dec 1;22(12):1129–42. doi: 10.1089/cmb.2015.0146 (PMC4663649; doi:10.1089/cmb.2015.0146)
Supplement: Supplemental data [file Supp_Figs1-4.pdf]

Supplementary Data

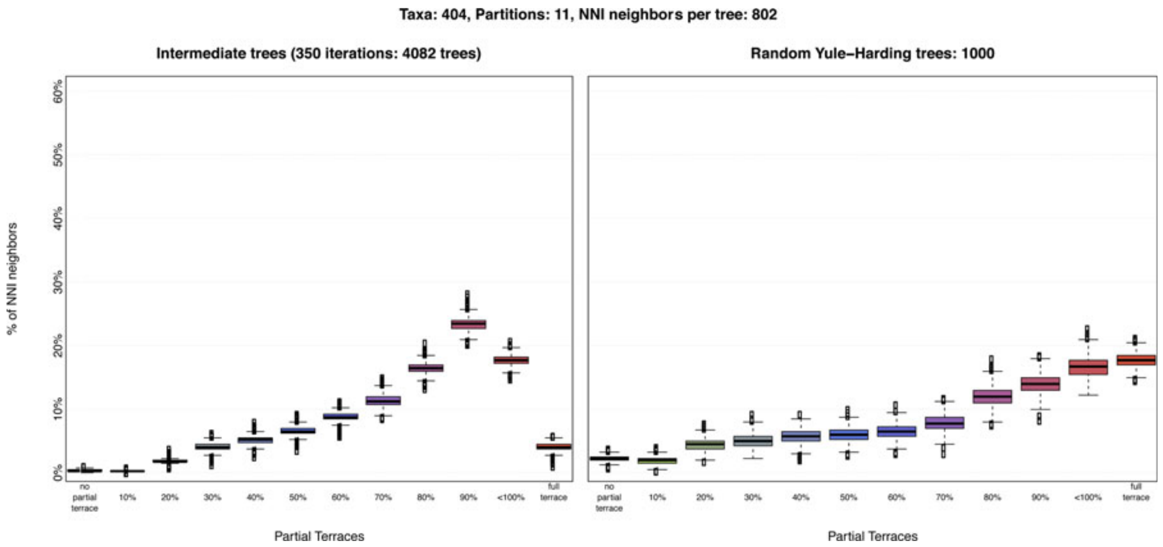

SUPPLEMENTARY FIG. S1. Analysis of NNI neighbourhood of intermediate and random trees for DNA4.

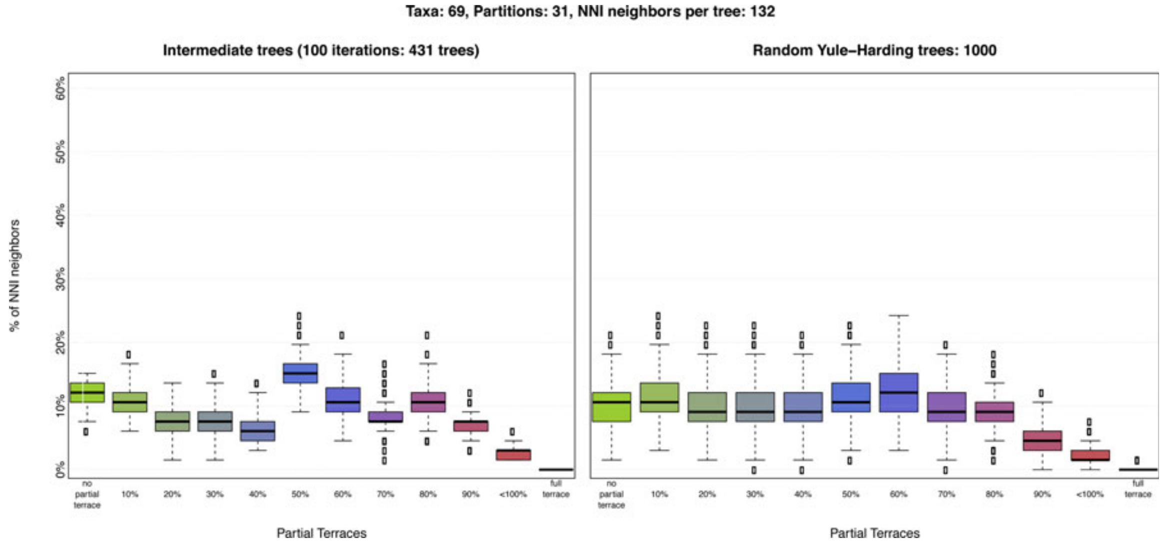

SUPPLEMENTARY FIG. S2. Analysis of NNI neighbourhood of intermediate and random trees for AA1.

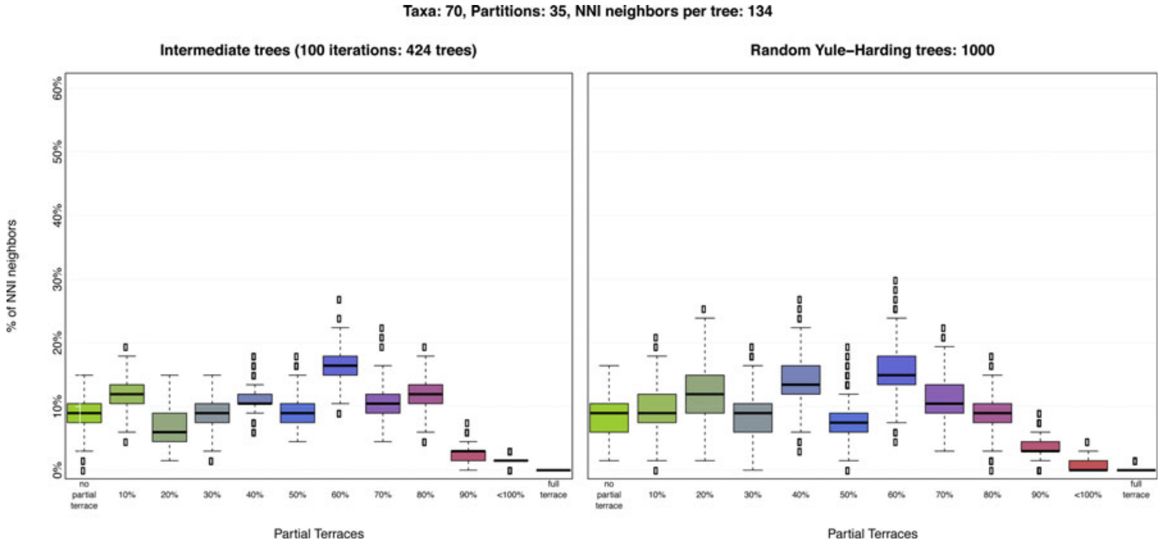

SUPPLEMENTARY FIG. S3. Analysis of NNI neighbourhood of intermediate and random trees for AA2.

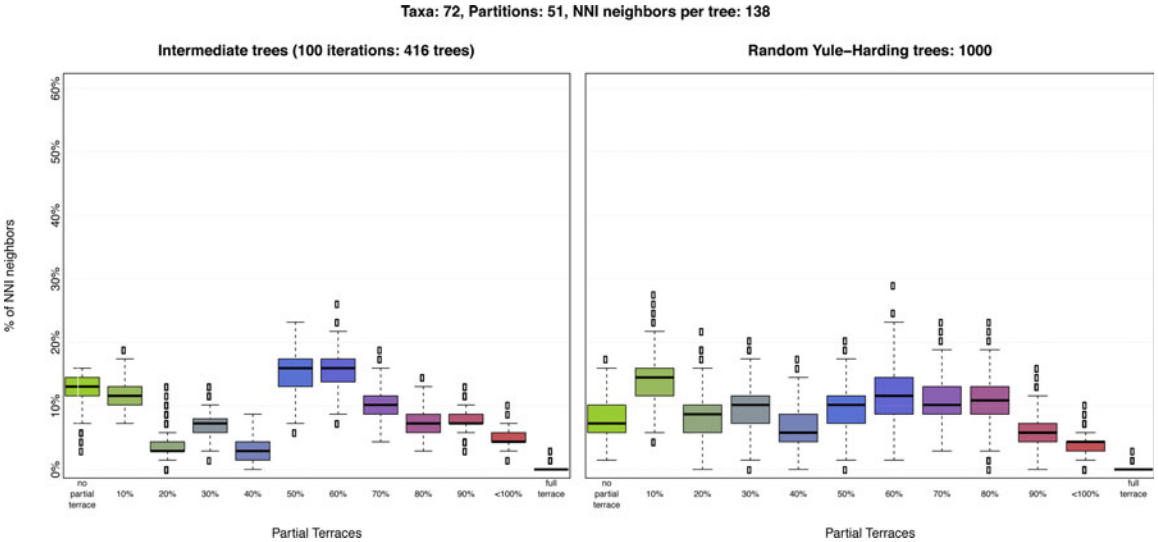

SUPPLEMENTARY FIG. S4. Analysis of NNI neighbourhood of intermediate and random trees for AA3.
